# Supplementary material for: Direct and indirect genetic effects of birthweight predisposition on child DNA methylation at birth
Source: Epigenetics. 2026 Apr 14;21(1):2654101. doi: 10.1080/15592294.2026.2654101 (PMC13081746; doi:10.1080/15592294.2026.2654101)

Figure S2. Marginal effects of maternal effect birthweight genetic predisposition depending on child sex

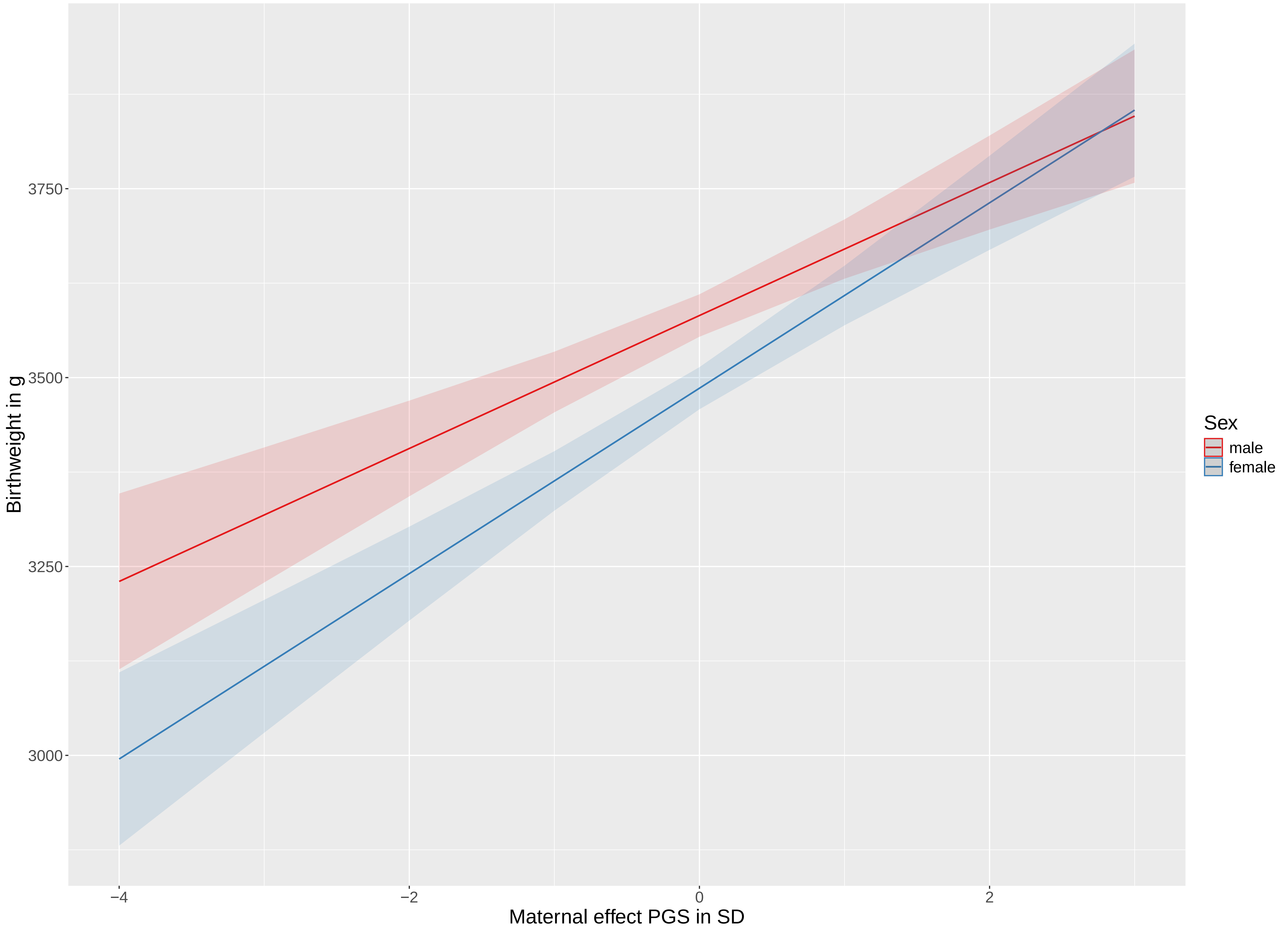

Supplement: figureS2_sex_int.pdf [file KEPI_A_2654101_SM8031.pdf]
